# Relational trajectories in families with parental mental illness: a grounded theory approach

**DOI:** 10.1186/s40359-020-00432-2

**Published:** 2020-07-01

**Authors:** Pamela Marie Patrick, Andrea E. Reupert, Louise A. McLean

**Affiliations:** 1 Monash University, 2/5 Florence Avenue, Clayton, VIC 3168 Australia; 2 Faculty of Education, Monash University, Clayton, Melbourne, VIC 3800 Australia

**Keywords:** Adult children of parents with mental illness, Qualitative research, Grounded theory analysis, Emerging adulthood, Intergenerational families, Relational trajectory

## Abstract

**Background:**

Adult children of parents with mental illness experience a myriad of complex emotions as they attempt to make meaning of the lived experiences of their parents. A crucial time for adult children is emerging adulthood, a time when they move away from their family of origin and establish their own identity and independence. Despite existing research that provides a static description of adult children’s lived experiences, the literature lacks an explanatory theory about the dynamic, relational processes that occur as adult children progress from one life stage to the next.

**Methods:**

The current study aimed to develop an explanatory theory of the relational trajectory that adult children might experience as they course through adulthood and parenthood over time. Semistructured interviews using grounded theory analysis were conducted with 10 adult children aged between 27 and 51 years old.

**Results:**

Three key phases within the Relational Trajectory Model (RTM) were identified: (i) confusion, (ii) contemplation, and (iii) reconciliation. By reflecting on their own parenting role, adult children were able to reach an evolved parental identity, with the majority of participants also making relationship reparations with their parents with mental illness. Parallels are drawn to theories of identity and intergenerational family systems to further explain and substantiate the processes encompassed within the RTM.

**Conclusion:**

Generating an explanatory theory serves as a potential guide for mental health professionals working with families with parental mental illness, by drawing attention to the intricacies of familial relationships and interpersonal ties.

## Background

In the United States of America (USA), an estimated 7.5 million adults with depression have at least one dependent child (i.e. 18 years or under) living with them [1]. Approximately 15 million children in USA live in households with parents who have either major or severe depression [1]. In other countries, prevalence rates of children living with a parent who has a mental illness are also high. The Netherlands, with a population of 16.8 million, has an estimated 17% of children under the age of 18 live with a parent with mental illness [2]. In Australia, researchers estimated through the triangulation of data from the Australian Bureau of Statistics, records from mental health providers and a community survey, that 23.3% of Australian children (i.e. one in five children) live in families where a parent has a mental illness [3]. Parental mental illness can pose a myriad of challenges for parents and children alike [4]. Children who have a parent with a mental illness are at significantly greater risk for multiple psychosocial problems, compared to those whose parents do not have a mental illness [5].

Children of parents with mental illness are susceptible to a range of genetic, environmental and social risk factors. Parental mental illness has been reported to adversely impact a child’s attachment relationships [6], expressive language development [7], incidence of school problems, including academic underperformance [8], and elevated interpersonal difficulties and behavioural issues [9]. Additionally, due to the stigma associated with mental illness, children may experience problems with shame, embarrassment and isolation [10]. Several studies have found that the aforementioned risk factors are strongly related to housing insecurity, unemployment, and financial difficulties [11]. The difficulty distinguishing the direct effects of parental mental illness (which depending on the illness may include suicidal ideation or delusions) from the indirect effects (e.g., marital disharmony, unemployment, impaired parenting) has been acknowledged given the synergistic nature of these elements [12, 13].

### Adult children of parents with mental illness

There is some suggestion in the literature that the challenges of living with a parent with mental illness may have an intergenerational bearing on future generations of children [14]. Termed ‘toxic inheritances’, the process through which distortions, silences and violence are passed down from one generation to the next, have been shown to exert serious intergenerational impacts on families living with parental mental illness [13]. Within family systems theory, ‘differentiation’ of self is vital for healthy development and the formation of intimate relationships both within and outside of the family unit [15]. Differentiation entails the ability to navigate between individuality and togetherness in a relationship system [16]. Evidence from a qualitative study of 13 children of parents with mental illness, suggests that these children may not have sufficient experiences of differentiation as loss of identity was found to be a central theme, and was postulated to be due to the limited opportunities for young people to be involved in self-defining activities [17]. Loss of self-identity has been linked to experiences of intense feelings of sadness, anger, regret and other powerful emotions [18]. A synthesis of qualitative findings from seven papers on adult children of parents with mental illness identified several other commonalities across participants’ narratives [19]. Across the papers of this review it was found that adult children discussed parental absence in their lives and parentification as common experiences. The lack of external family support, feelings of isolation, and difficulty trusting others posed additional challenges for the adult children included in the review. Other experiences included grief, worry, emotional blocking and a maladaptive sense of self where adult children viewed themselves as being “defective” or blaming themselves for things that went wrong in their families. At the same time, some described a sense of hope, or opportunity for growth believing that they could deal with almost anything [19]. However, the experiences of adult children remain ambiguous, as several of these studies described family experiences without differentiating amongst the narratives of siblings, adult children and other relatives. Furthermore, as acknowledged by Murphy and colleagues, the studies within the meta-synthesis included dated research, which underscores the need for contemporary research in this area [19].

Longitudinal studies have explored the impact of negative or stressful experiences (e.g., parental mental illness, substance abuse, parental divorce, physical or sexual child abuse) on the attachment of adult children [20]. These studies found that individuals who experienced a chaotic or stressful life experience had a higher propensity to transition from secure to insecure attachment [13, 20]. However, protective factors, such as reflecting and making meaning of a negative life event were helpful when moving from an insecure to a secure attachment pattern [13].

In relation to their own parenting roles, adult children were noted to experience parenting anxieties due to a perceived lack of a parenting reference point [21]. This was further compounded by the difficulties of having to parent in the absence of external family support upon which they could rely [22]. Additionally, given their experiences growing up with a parent with mental illness, some adult children found it challenging to relate to their child’s everyday problems such as friendship issues, given their own vastly different experiences growing up with a parent with mental illness [22].

The aforementioned themes suggest ongoing challenges and relationship difficulties for adult children, both in their family-of-origin and family-of-procreation [19, 23, 24]. These children are more likely to encounter strained family relationships, marital problems and family breakdown than children with non-affected parents [25]. Over time, children who are exposed to parental mental illness are also at risk of their own psychopathology in adult life [26, 27]. Collectively, research suggests that parental mental illness is likely to influence their children’s development as well as exert a significant influence on how their children will, in turn, cope with new social roles such as becoming a spouse or a parent.

There are a number of limitations in previous research regarding adult children whose parents have a mental illness. First, existing research offers a static one-time account of different life stages in an adult child’s life (e.g., childhood or parenthood). Though valuable, descriptions of singular phases or time points only provide ‘one piece of the puzzle’. Thus, previous research falls short in mapping out the relational path that adult children commonly encounter as they transition from one developmental stage to the next. A cohesive understanding of a child’s emotional processes, and parent-adult child dynamics across time may allow for the development of appropriate prevention or intervention methods that can be delivered at different times [28]. Second, there is a lack of a unifying theory to guide families and practitioners in understanding relationship dynamics that commonly occur in families with parental mental illness. The absence of a conceptual framework stifles the ability for research advances in this area, specifically in the development of theoretically based interventions and support services aimed at this group [29]. The present study used in-depth, semistructured interviews and grounded theory strategies to answer an under investigated research question: What are the relational processes for adult children as they progress through various life stages?

## Method

### Grounded theory approach

This study employed a constructivist Grounded Theory (GT) approach [30] to develop an explanatory model of the relational processes experienced by adult children. Qualitative enquiry offers a means of gaining depth and clarity about participants’ experiences, particularly in underdeveloped areas of research, to create data-based theories [31, 32]. GT, in particular, is a qualitative approach that has been utilised due to its usefulness in exploring family processes [33]. While constructivist GT is similar to earlier methods of GT, it differs in several ways including: (i) the assumption of a relativist epistemology, (ii) acknowledgement of researcher and participants’ multiple roles and realities, (iii) adoption of a reflexive stance towards background, values, relationship with research participants and representations of them and (iv) situation of research within historical, social and situational conditions [30]. Realities within constructivist GT are constructed by individuals ‘under the influence of a variety of social and cultural factors that lead to shared constructions’ between research and participants [34].

GT differs in important ways from other qualitative designs, such as phenomenology and ethnography. Phenomenology is rooted in understanding the essence of experiences while ethnography is embedded in interpreting stories within a cultural context or between culture-sharing groups. In comparison, GT seeks to develop a theory that is grounded in data [35], thus serving as the method of choice for this project. In GT, purposive sampling, data collection, and data analyses occur concurrently [36]. In purposive sampling, study participants are intentionally sought to help researchers explore specific ideas about the developing theory [37]. Rather than generalising results from a sample to a broader population, the goal of grounded theory is to sample *experiences* broadly in order to identify concepts and contextual factors that are relevant to understanding the phenomenon of interest [33]. Data are collected in GT studies until saturation is reached. Saturation is conventionally defined as “the point in the research when all of the concepts are well defined and explained” [37]. In qualitative methodologies, such as GT, it is essential to view saturation in terms of sample adequacy, data quality and variability of relevant events more than the number of participants [38]. Although new information will always be gained when gathering data from additional participants, in the present study, recruitment stopped when the research team judged that the inductive concepts were developed sufficiently and the relationships between concepts were clear. The research team reached consensus following several rounds of discussion, coding and memo-taking. Although there are multiple approaches to conducting GT, the above process reflects the broad goals of these approaches [33].

### Ethics

The study was approved by the Monash University Human Research Ethics Committee (project number: 10598). All participants received information sheets and signed an informed consent form prior to participation. Permission for audio recording was also sought prior to each interview for the purposes of transcription. Participants were informed of their right to terminate participation during the interview itself or withdraw their data (after the interview) at any point in time prior to data analysis. Participants were also informed of their right to not answer any questions posed by the interviewer. Participants received a list of counselling helplines if they felt distressed during or after the interview. Identifying information within transcripts was replaced to ensure anonymity and a copy was also sent to participants for verification purposes. Transcripts were stored in a password-protected laptop issued by the University, accessible by the first author only.

### Inclusion criteria

The inclusion criteria specified that adult children needed to: (i) have/had at least one parent meet the above mentioned criteria of mental illness and (ii) have at least one dependent child between 0 and 18 years of age, and (iii) be residing in Australia. Participants were purposefully sampled and were heterogeneous in terms of their ages. Among the 10 participants, eight interviews were conducted via telephone and two interviews were done in-person.

### Recruitment

Adult children of parents with mental illness were recruited via electronic flyers sent to mental health practitioners across Australia. Similar recruitment methods were used to recruit participants at mental health and nonprofit organisations that supported carers and families of individuals with mental health issues. For the purpose of this research, ‘parents with a mental illness’ was defined as individuals affected by at least one psychiatric disability and/or who reported to receive ongoing or past psychiatric treatment (e.g., medication, counselling, psychological intervention) as an indication of experiencing mental health issues [39]. Electronic flyers contained a brief overview of the research and inclusion criteria.

### Participant demographics

This paper forms part of a larger study, where an interview schedule was designed to investigate different experiences. Ten adult children (9 females, 1 male) participated in one-on-one interviews, which included questions that tapped on participants’ life stages (i.e. childhood, adulthood and parenthood). Another paper has been published that presented participants’ parenting experiences [22]. The current paper is different and reports on the relational trajectories experienced by adult children who lived with childhood parental mental illness. Participants’ age ranged from 27 to 51 years old (*M* = 40.30, *SD* = 6.83). Seven participants identified as Caucasian, one as Aboriginal Australian, and another of Asian decent. One participant did not specify an ethnicity. Nine participants reported that their parent had received a formal diagnosis from a mental health practitioner. One participant, however, shared that her mother had initially received several sessions of counselling for a narcissistic personality disorder query. However, as her mother subsequently refused to seek ongoing professional help, a formal diagnosis was not obtained (denoted by an asterisk in Table 1). Additional participant details are included in Table 1.

**Table 1:** Adult child and parent demographics

Pseudonym	Participant Gender	Participant History of Mental Illness (Y/N)	Parent with Mental Illness	Parent’s Mental Illness
Lionel	Male	Y	Father	Depression Anxiety
Claudia	Female	Y	Mother	Narcissistic personality disorder *
Gabrielle	Female	Y	Father	Depression Anxiety Alcohol addiction
Anna	Female	Y	Father	Bipolar disorder
Suzanne	Female	Y	Mother	Depression Narcissistic personality disorder Alcohol addiction
Laura	Female	N	Mother	Bipolar disorder
Tanya	Female	Y	Mother	Paranoid schizophrenia, Post-natal depression Post-natal psychosis
Jenny	Female	Y	Father	Posttraumatic stress disorder Bipolar disorder
Katie	Female	N	Father	Paranoid schizophrenia
Evelyn	Female	N	Father	Bipolar disorder Manic depression Obsessive Compulsive Disorder

Formal diagnosis was not obtained due to lack of treatment adherence

### Semi-structured interview schedule

The authors developed an interview schedule (refer to Additional file 1) that was based on the research question, their clinician and research experience and existing literature. To aid participants’ recounts, questions were ordered in a chronological order, commencing with experiences about childhood, followed by adulthood and then parenthood. A script for the semi-structured interview was also developed and trialled amongst the research team prior to the commencement of interviews. At the start of each interview, participants were provided with a brief overview of the purpose of the study and the option of withdrawing or refraining from answering any question, if they chose to. The interviews started with an open-ended question: “How would you describe your relationship towards your parent who had a mental illness when you were a child?” after this question, follow-up questions such as: “can you give me some examples of this?” were asked to encourage further elaboration. The interviews lasted between 58 and 101 min (*M* = 80.00).

### Data analysis

All interviews were audio-recorded following participants’ consent and transcribed verbatim by the first author, including laughter and speech pauses (e.g., ‘uhm’ and ‘arhs’). However, as the majority of interviews were done via telephone, non-verbal data were not documented. Data management and analysis were managed with QSR NVivo 12 software. The interviewer and research team were well acquainted with the general literature on parental mental illness in families. The team consisted of women only, with clinical as well as research expertise in this area. A number of steps were undertaken to ensure trustworthiness of findings including prolonged engagement with participants and peer debriefing amongst the research team to confirm emerging concepts and categories. Member checks were also conducted, which involved sharing with each participant a verbatim transcript of their interview and inviting them to amend or delete any aspect of their transcript they believed was not representative of their intended meaning, or to add anything they considered important [40, 41]. Only one participant made minor alterations.

Grounded theory is a nonlinear methodology that allows for different themes and ideas to be identified during analysis through the process of creating, comparing, and contrasting categories within the data [42, 43]. In line with constant comparative analysis, data collection and analysis occurred concurrently [44]. Data analysis commenced with a line-by-line review of the transcripts. Initial codes with similar themes and underlying principles were combined into broader overarching categories [45]. Following the first few interviews, an initial grounded theory emerged from the data. These codes were then used to code subsequent interviews (from new participants). Constant comparison within and between participants led to a more defined model. Saturation checks were completed by reviewing each participant’s data for the presence of identified overarching categories, complete with descriptions and pertinent examples for each concept [46]. The last phase of analysis focused on clarifying the relationships between categories and codes as well as testing the boundaries of those codes by analysing negative cases and constant comparison. In comparing negative cases, it was apparent that some participants did not transition through the full relational trajectory but rather experienced “differential pathways”. Overall, coding focused on understanding the purpose of each category and the sequential flow of participants through the model [29].

Common approaches, such as verification and self-correction have been suggested practice to enhance the reliability and validity of qualitative analysis [47]. In particular, Morse and colleagues purport that in order to ensure rigor of qualitative projects, there is a need for several considerations. Methodological coherence, which is fit between research question and method, sample appropriateness (i.e. selecting representative participants to address the research questions) paired with concurrent data collection and analysis. These further enabled theoretical thinking (i.e., corroborating of new data with collected data) and theory development (i.e., evaluate findings in context of current knowledge) [47]. These approaches were adopted in the current study using GT to develop an inductive theory, where limited research exists [29]. GT was also beneficial in allowing provisions for constant comparison including reviewing previously collected interviews for any new concepts identified. Concepts that developed through the data were also checked with other participants through open-ended probes to avoid bias. Thereafter, a literature review was undertaken to check consistency between the model and current knowledge [29]. This review was conducted by drawing on literature in the Psychinfo, Proquest, PubMed and Google scholar databases, with key terms such as ‘adult children’, ‘offspring of parents with mental illness’, grown-up children’, and ‘parental mental illness’.

## Results

### The relational trajectory model

**Fig. 1:**
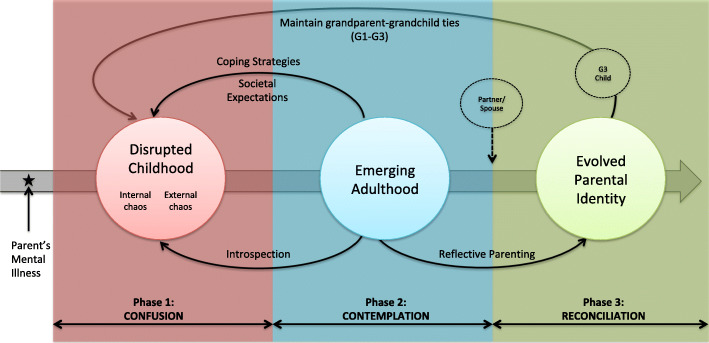
Graphical representation of Relational Trajectory Model. The Relational Trajectory Model (RTM) is an explanatory model derived using grounded theory analysis of data gathered from ten adult children of parents with mental illness. The model provides an overarching process through which adult children’s relational roles are influenced and shaped as a result of their lived experiences of growing up with a parent with a mental illness. Based on the data, adult children underwent three primary mental states (i.e. confusion, contemplation and reconciliation) that consequently influenced relationships, particularly with their own children and parents. The subprocesses identified within the RTM can be either forward or backward influencing. In other words, subprocesses have the ability to influence meaning making of past experiences (i.e., backward-influenci4dqwng) as well as transform future experiences (i.e., forward-influencing). xsThe RTM provides a preliminary theoretical model that moves beyond description, to the explanation of how adult children transition from one life phase to the next, within the context of their parents’ mental illness. The RTM was developed by authors of this research paper

From the data collected, the authors identified a three-phase process that was subsequently developed into the Relational Trajectory Model (RTM).^1^ The RTM was developed as a preliminary model to explain the varying phases adult children undergo as a way of coming to terms with parental mental illness and how they subsequently responded and adjusted to relational roles, both in their families of origin and in their families of procreation. The objective of the RTM model is to move beyond prescriptive literature describing adult children’s experiences, and to explain how and why some adult children manage to progress to relational growth. Based on the data, adult children underwent three primary mental states (i.e. confusion, contemplation and reconciliation) that consequently influenced relationships, particularly with their own children and parents (see Fig. 1). These mental states corresponded with key life stages encountered by adult children in this study (i.e. disrupted childhood, emerging adulthood and evolved parental identity). Although the prototypical progression within the RTM model is linear, in that participants largely progress through mental states in the order presented in Fig. 1, subprocesses (i.e. indicated by curved black arrows) can be either forward or backward influencing. In other words, in any given phase, subprocesses in the RTM are postulated to have the ability to influence meaning making of past experiences (i.e., backward-influencing) as well as transform future experiences (i.e., forward-influencing). The RTM and its subprocess are described in the following section and further illustrated through participants’ quotes.

### Phase one – confusion

The first phase of the model, labelled ***‘confusion’***, denotes the overall cognitive state of most participants during their growing years. Participants described experiences from their growing years that could be classified into ‘internal chaos’ and ‘external chaos’, both of whichs contributed to the overarching sense of confusion. Internal chaos describes the cognitive processes taking place at an intrapersonal level. For instance, lack of open communication and knowledge about their parent’s mental illness throughout their childhood often left participants with more questions than answers. Due to their parent’s mental illness, some participants also described being unable to relate to their peers, which inadvertently fostered feelings of isolation and loneliness. The following quote depicts a participant’s narrative of internal chaos:

*“I do remember that there was a visit from somebody, I don’t know where from, possibly from child protection but all I can remember was them getting me to draw a picture of a body and telling them about the “rude parts” and what they were called. I don’t know what that was… a bit random but no one ever explained about the illness and what was gonna happen. And there was a sense of always “walking on eggshells” – just not knowing when the next episode will happen…” (Laura).*

Conversely, external chaos was used to describe situations that commonly arose as a result of living with a parent with mental illness, external to the participant. Domestic violence, parental separation, parental mood fluctuations and unpredictability or volatility in the home environment were examples of ‘external chaos’ commonly reported by participants, as one participant shared:

*“I think obviously when she [mother] developed the psychosis that was quite scary because she became quite violent and destructive and was throwing things at me and destroying things and saying things that were quite hurtful. And because I think I didn’t really understand what was going on, it created an even bigger distance and I guess a lack of trust and not knowing when this person was suddenly gonna go crazy again. So it was kinda hard.” (Tanya).*

### Phase two – contemplation

Phase two of the model, termed ***‘contemplation’***, signifies participants’ transition into emerging adulthood, which is characterised as a period of time between an individual’s first entry into adulthood and middle adulthood. For most, this middle phase was where many of the cognitive subprocesses began to take place. Introspection captured participants’ reflective practices trying to make meaning of their childhood lived experiences, whilst forming their own identities and contemplating the type of relationship they wanted to have with their parents. Participants also acknowledged that through introspection, they were also able to see the strengths they had attained despite familial adversities:

*“I think there’s also an appreciation of the idea, had I not gone through what I’d gone through, then perhaps I might not have been as aware, I guess, of how different – these marginalised experiences can have a very profound impact on the lives of adults, or children… I guess that helps me cope and knowing that it’s actually resulted in a strength – in a personal strength for me.” (Anna).*

Within the contemplation phase, participants also acknowledged two other aspects that facilitated meaning making and coming to terms with their childhood experiences. The subprocess labelled ‘coping strategies’, consists of a number of coping skills utilised by participants to come to terms with, and enable adaptive coping in regard to their parent’s mental illness. Several coping strategies were mentioned by participants, including seeking social supports, setting boundaries, accessing mental health services, acquiring knowledge about their parent’s mental illness and turning to religion. The use of some of these coping strategies are further illustrated in the following participants’ narratives:

*Social Support – “There are lots of things that we [siblings] do strategically. Like my sisters and I are often talking about what’s going on with mum and we all help each other and if she’s got an issue that she’s worried about, we’d warn each other about what’s happening because you can offend her just so easily. So I think the three of us have to work really close together to monitor how she’s going because it is so easy to say something… she [mother] gets really worried about what we’re going to say and what might happen.” (Evelyn).*

*Religion – “I attended a Christian university and there was a sermon about forgiveness…my father never really hurt me, but there was no interaction, so I tried to forgive him and I tried to reach out to him. It was not much but I just tried to talk to him.” (Claudia).*

*Setting boundaries – “… have really firm boundaries and to say: “nope, I’m not going to be a part of that. I’m outside of that” and that’s how I’ve kinda maintained my own mental health, because if I keep hoping and wasting all this emotional energy on something that’s totally out of my control, then you know, I can be friendly, I can do everything but I’m not going to change this person, you know... so I just need to be realistic about that.” (Tanya).*

Although the aforementioned coping skills could be perceived as contributing to growth in parent-child relationships, for a small subset of participants, emotional numbing or blocking was described as a necessary coping skill. Some participants intentionally severed ties with their parent as they preferred not to subject themselves to their parent’s emotional volatility, and at times, verbal abuse. For others, the fear, disappointment and hardship encountered during their childhood outweighed any desire or intentions for relationship reparations. The other way in which adult children maintained an ongoing relationship with their parents was through the subprocess labelled ‘societal expectations’. Societal expectations are descriptive of participants who described the relationship shared with their parent as one born out of obligation – driven by deep-seated religious beliefs or cultural expectations, such as filial piety, as shared by one participant:

*“I believe that I do my duty. I do the bare civilities of you know, Christmas, Birthdays… I might invite her [mother] to school events or dance displays but she’s not a close part of my life, because she still does things and says things that are not trustworthy.” (Tanya).*

Within phase two, ‘reflective parenting’ was a forward-influencing subprocess that encapsulated adult children’s reflections specific to their subsequent role as parents. This subprocess typically occurred when participants were contemplating starting families of their own or during pregnancy itself, and was conveyed as follows:

*“I remember when I was pregnant with my first child, I sat down and wrote a list of things that I loved, particularly in my mum and how she raised us and things that I loved about my childhood and my parents, and the things that I didn’t want to repeat. So I hope that in the things that I’ve found really comforting that I have continued those things in my own parenting but the other things, I’ve deliberately tried to change…I think I am very different to my parents but I don’t want to reject everything that they did because there were some things that were really good. I’m certainly much more connected to my children than I ever have been to my parents and that’s something that I hope continues right through their lives.” (Katie).*

For mothers in particular, feelings of uncertainty about parenthood began to arise as many felt they lacked a parenting reference point. The feeling of uncertainty pre-empted some participants’ reflective parenting process. Reflective parenting was done in a deliberate manner for those participants who were mothers, to process the kind of parenting they had received, and the kind of parent they envisioned themselves as being. For several participants, the concept of ‘oppositional parenting’ – parenting in opposite ways to one’s own parent, emerged. Oppositional parenting was described as a deliberate way for these participants to engage in parenting differently from their parents and to make their unique parenting contributions with their children. For some participants, their spouse or partner also influenced this reflective parenting process by offering alternate perspectives or ways of upbringing that were different to what participants themselves were familiar with and experienced during their childhood.

*“I’ve also sort out ideas and good ways to do it [parenting] and sort of done that attachment-based parenting but I haven’t taken this uncritically from my childhood and mirrored it. I have thought about what I liked and my partner’s had some contributions from his family as well … I feel like I’ve been thoughtful about how to parent.” (Jenny).*

### Phase three – reconciliation

The third phase of the model, termed ***‘reconciliation’*** occurred when adult children became parents themselves. Reflective parenting was described as a key process in participants’ attainment of an evolved parental identity, as participants attempted to parent in different ways from their parent(s) or to parent in an evolved version of the parenting they had received in their growing years, as one participant explains:

*“I hope I’m the “good one” – authoritative parent. I think I spend a lot of time talking with my children… I try to be very involved but I’m also mindful of not trying to be overly indulgent because I felt like that’s something my mum did, and as a result I didn’t end up respecting my mum. She [mother] thought parenting was “give the kids what they want and need”, whereas growing up with that, I felt like I just needed her [referring to mother’s emotional presence].” (Anna).*

For many participants, becoming a parent was a central component of their identity and helped them acquire new insights and appreciation of their own parent. Adult children acknowledged that parenting was challenging in and of itself without the added challenge of dealing with a mental illness. Adult children were also keen for their children to know their grandparents and sought ways to ensure grandparent-grandchild ties were maintained. For some participants, bonding over grandchild stories or parenting experiences was seen as another avenue through which relationships with their parents was strengthened. The following narrative highlights how one participant tries to foster positive relationships between her daughter and mother:

*“For my daughter, you know, she only has one grandmother, so I guess I try to help gain some sort of positive sense of her grandmother.” (Tanya).*

### Different paths

The RTM and its subprocesses helps to explain how the majority of adult children in this study internally processed their lived experiences. Through the various subprocesses, all participants discussed an eventual attainment of an evolved parental identity – an identity that was achieved through reflection and conscious decision-making about the type of relationships they wished to foster with their own children. In most cases, the subprocesses seen within the RTM also paved the way for relationship reparations between adult children and their parent with mental illness, albeit with some exceptions. For some, the relational trajectory appeared to take on a different path than the one prescribed and occurred for two main reasons. First, one participant described not being able to forgive her parent for the emotional pain and volatility she experienced in her childhood. Therefore, in an effort to protect her own mental wellbeing and prevent ongoing hurt, she intentionally chose to cease contact with her parent. Second, though some participants progressed through the phases of RTM, external influences made it challenging for them to sustain ties with their parent. One participant described their parent as being harsh and critical not only of them but also with their grandchildren, while another shared about her mother’s compulsive need to lie, which ultimately hampered their ability to form a trusting relationship with the parent with mental illness. Therefore, external factors, particularly the nature and severity of the parent’s mental illness was a crucial component that determined the quality of relationship between an adult child and their parent with mental illness. For these participants, the lack of ties with their parent also meant that the subprocesses pertaining to grandparent-grandchild was absent.

## Discussion

The purpose of GT is to develop an explanatory model of a human experience by grounding theory within data generated from an appropriate sample [47]. This resulting RTM provides a preliminary theoretical model that moves beyond description, to the explanation of how adult children transition from one life phase to the next, within the context of their parents’ mental illness. Adult children may move through three broad phases, from disrupted childhood, emerging adulthood and lastly to an evolved parental identity. Each phase within the RTM is marked by distinct mental states, which served as a way for adult children to confer meaning and to interpret their lived experiences. Conferring meaning is a basic human motivation. In particular, research on individuals who have undergone trauma and violated their basic life assumptions tend to engage more in introspection and conferring meaning as a way of making sense of their lived experiences [48]. Descriptively, several of the subprocesses in this model overlapped with underlying principles from other theories, such as, identity theory, intergenerational family systems theory and determinants of parenting. .

### Identity theory

The emerging adulthood phase could be viewed as the most crucial point within the RTM, as adult children engage in internal thought processes as a way of making sense of their past and shaping their future. Arnett coined the term *emerging adulthood* to define the period of 18 to 25 years of age, which was distinct from adolescence and young adulthood [49]. The phase is characterised as a prolonged phase of exploration and change, and as a time before individuals make their way to adult roles and responsibilities [49]. Although many changes accompany emerging adulthood, it is characterised primarily by a “recentering” of relationships [50]. In the present study, the identity of being an adult child of a parent with mental illness served as a primary motivator for participants to engage in introspection and reflective parenting. The latter process was particularly salient when participants thought about starting families of their own, and/or for female participants, during their pregnancy. The emerging adulthood phase embodied a time point where participants sought to create identities of their own by reinventing themselves and contemplated starting families of their own [51]. For majority of participants, the need to reinvent oneself was fuelled by years of experiencing confusion, distress, and being personally overwhelmed living with parental mental illness [51], which is characterised by the disrupted childhood phase in the RTM. Hence, for most participants emerging adulthood served as a time for self-reflection.

Within self-categorization theory, identity is assumed to be fluid and dynamic [52, 53]. As participants embarked on their self-reflections and entered a new life stage of emerging adulthood, their salient identity also transitioned from being a dependent child of a parent with mental illness to one of independence and autonomy. Although the interviews took place at a single time point, data demonstrated that participants’ identity was continually evolving and changing, as they adopted various relational roles, such as becoming one’s spouse or parent. This was evident in participants’ descriptions of themselves over time, and the ways in which they connected with others including disconnecting from unhealthy relationships and becoming more discerning or alternatively, learning to trust [52].

### Intergenerational family systems theory

Emerging adulthood is characterised by a young individual leaving home and is viewed as a critical window from the perspective of intergenerational family systems theory [54, 55]. Within intergenerational family theory, Personal Authority in the Family System (PAFS) can be seen as a new family developmental stage that incorporates both individual and family life cycle stages [56]. The central focus of this stage is renegotiation and the termination of the hierarchical power boundary between the young adult and parents. The power inequity between a parent and child is sustained when children are younger and dependent on their parent. Developmentalists, however, indicate that most children attain PAFS between the ages of 30 and 45 as adult children leave their parents’ home and grow in their autonomy and independence [15]. Hence, as PAFS occurs, it is likely to bring about a certain degree of individuation amongst adult children, who may use that as a way of separating their identity from their parent’s mental illness.

PAFS refers to the degree of psychological health depicted by the degree of individuation and intimacy within the family of origin [56]. Within this context, individuation is regarded as the ability to function in intimate relationships, without having to assume a disproportionate amount of responsibilities for family members. Intimacy implies relational closeness with distinct boundaries to the self that may be initiated or terminated by choice. Applying the PAFS concept to the RTM model, it would appear that adult children in the current study strived for individuation by reflecting and executing new ways of parenting and forming family rituals and practices of their own. Conversely, intimacy for adult children predominantly occurred as a result of societal expectations and by employing various adaptive coping strategies. For some participants, the societal expectation that it is a child’s responsibility to care for a parent in their old age was instrumental in maintaining ties with their parents. For others, it was coping strategies, such as religious beliefs, filial piety [57], felt obligation [58] and a desire for their children to know their grandparents, which served as the bridge for ongoing ties between an adult child and their parent. Felt obligation and filial piety, in particular, have important implications for parent-child relationships in adulthood. A sense of indebtedness to parents in adulthood and duties performed throughout one’s life are seen as a form of repayment for parental sacrifices [58, 59], appeared to be a key driving factor for sustained relationships between adult children and their parents.

A smaller subset of participants, however, reported that even though initial attempts were made at sustaining a relationship with their parent, their parents’ emotional volatility particularly criticism towards grandchildren served as a breaking point resulting in severed ties between parent, the adult child and grandchildren. Decisions to trust or not to trust, and the degree of trust to invest in, are fundamental elements in any relationship [60]. To trust another person involves some notion of risk to the self, as a person must give of themselves, prior to knowing how the other person may react or behave with them in return [51]. PAFS includes individuals’ abilities to know and direct their own thoughts and feelings and their ability to connect and interact emotionally with other people [15]. As such, based on the PAFS, one chooses with whom to develop closeness, love, trust, commitment and mutual respect while maintaining individuation [15]. For some adult children, external factors, such as the severity of the parent’s mental illness, parent’s emotional volatility or their personal coping mechanisms were some reasons why participants described having an alternate pathway other than the prototypical RTM put forth in this study.

### Determinants of parenting

The determinants of parenting model posit that multiple factors interact and influence parental behaviour [61]. The three major determinants, namely, psychological well-being of the parent, characteristics of the child and contextual sources of stress and support are areas that jointly determine parental competence [61]. This notion is supported by the results of this research study, which identified both the context (e.g., spousal influence) and process categories (e.g., introspection) that interact and influence the experience of adult children of parents with mental illness [28]. It was further reasoned that the parenting system is buffered against threats that are derived from weaknesses in any single source or determinant [61]. As adult children embarked on the process of self-recovery and eventually entered parenthood, adult children sought emotional connection and belonging to other people, which the majority of participants could not obtain from their parent with mental illness. An evolved parental identity occurred when adult children perceived their parental identity as having evolved from their past experiences. It was also directly related to the adult child’s ability to grow, change, progress, advance or move forward from the parental role model that they experienced in childhood [28]. Therefore, how adult children responded to the challenges associated with parenthood and evaluated themselves as parents influenced their ability to achieve an evolved parental identity [28].

Also influential was the closeness that adult children shared with their spouse or partner. Being able to hear a different perspective – a narrative of a more “normal” childhood from their partner or spouse was found to benefit the overall parental identity formed by adult children in the current study. Through evolution from their lived experiences, adult children in the current study described feeling a strong identity towards parenthood, which allowed them to feel more successful in their role as parents. Likewise, previous research has found that as an adult child transitioned to parenthood, comparisons of oneself to their parent added new meaning and value to their role as parents [28]. Additionally, adult children who are capable of forming connections, obtaining support, controlling the degree of influence exerted by their family of origin, and experiencing satisfying relationships with their spouse or partner may feel more prepared to manage the demands of parenthood [28].

### Variances within the RTM

This research aimed to examine the relational trajectory for adult children of parents with mental illness. The RTM examines a generational tie that is twofold: on the one hand, the relationship adult children share with their family of origin, specifically the parent with mental illness, and on the other, the bond adult children share with their own offspring. However, it became clear during data analysis that an adult child’s process was partly influenced by the severity and nature of their parent’s mental illness and the degree to which adult children were able to objectify and distinguish symptoms of their parent’s mental illness from their parent. These variances signify the challenges in documenting an adult child’s experience and trajectory fully, due to the dynamic nature of family interactions. This model also does not account for adult children, who because of their lived experiences made a conscious choice not to have families of their own. Previous research has highlighted that the fear of heredity of mental illness and difficulty connecting with others have deterred adult children from entering committed relationships and having families of their own [21].

### Limitations and directions for future research

The vast majority of participants in this study were females and mothers and therefore findings may not necessarily translate to the relational trajectories experienced by male adult children. Parenting research indicates that fathers have their unique needs and experiences distinct from mothers [62]. Additionally, more than half of female participants were in helping professions themselves, which may have inadvertently contributed to their reflective nature. Furthermore, seven participants had a history of mental illness themselves and sought professional help, which could have enhanced their ability to manage their emotions and to engage in reflective practices. Given this, further research is necessary to examine if the identified pathways in the current model apply or are different to other subgroups of adult children (e.g., adult children who are not parents themselves or adult children with no history of mental illness). Future research could also examine how the proposed RTM can be applied as a toolkit for service providers to better understand and gain insights into the complex thought processes that adult children undergo, in order to better support their needs. Further research is also needed to expand current understanding on the deviated pathways encountered by some adult children. An appreciation of how and why these deviated pathways occur can help mental health practitioners in better grasping the multidimensional facets of family relationships within the context of parental mental illness. Even if adult children have made a conscious decision to cease contact with their parent, it is vital that service providers respect their decision but also look into ways to support adult children as a way of buffering the effects of the lack of family or informal social supports. Future research efforts could also examine the nature of fluidity or bi-directionality between phases presented in the RTM. Although participants’ narratives in the current study did not allude to fluidity between phases, it may be premature to assume this to be the rule rather than the exception, given the small sample size. Lastly, it is important for future research to replicate, verify and if necessary modify this model, particularly in its applicability over a longitudinal period. Given the infancy of research in this area, the aforementioned research directions could be imperative in expanding understanding about adult children and their experiences from a developmental and lifespan perspective. Grounded theory, as a method, allows for flexibility and adjustment, which becomes advantageous as research continues to evolve and transpire [63].

### Clinical implications

The RTM has a number of implications for service providers working with adult children and their families impacted by parental mental illness. Broadly, the RTM confirms that many adult children process their lived experience as a means of adjusting to adulthood and parenthood and this processing may be capitalised during therapy. It is also evident that adult children have unique needs that have to be met. As individuals who may lack role models of appropriate parenting, it is vital that provisions are made available for adult children to address and enhance their parenting knowledge in areas they perceive needing more support with. Although the majority of participants found their internal thought processes to be helpful in relationship reparations with their parents, this was not true for every adult child in the current study. For some, the continuing efforts to connect with their parent were not reciprocated, eventually leading to severed ties. This in itself could leave an adult child feeling increasingly vulnerable and emotionally dejected. Hence, it important for mental health practitioners to be aware of the challenges adult children face and provide contexts for their feelings to be expressed and validated. Supporting families with mental illness is not an exclusive role for specialist mental health services, but for all health and social care professionals. Providing a safe space where adult children can raise their concerns or worries can offer reprieve to the anxiety and concerns often experienced by adult children.

## Conclusion

The main goal of this grounded theory research was to provide a synthesis of knowledge pertaining to the relational trajectory that adult children commonly experience as they progress from one developmental stage to the next. The paper highlights elements of personal coping, internal thought processes and the role of other family members, which in most cases had the capacity to mend parent-adult child dynamics that were often turbulent to begin with. This model provides a framework for mental health clinicians and future researchers to be informed about the emotional complexities faced by adult children thus increasing the potential for targeted interventions and related supports.

## Supplementary information

**Additional file 1.** Interview schedule.

## Data Availability

The datasets generated and/or analysed during the current study are not publicly available due the sensitive nature of narratives shared that may possibly disclose participants’ identities, but are available from the corresponding author on reasonable request.

## Abbreviations

RTM: Relational Trajectory Model
GT: Grounded Theory
PAFS: Personal Authority in the Family System

## Footnotes

1 The RTM shown in figure 1 was developed by authors based on the data set derived from research participants in the current study.
