# Supplementary material for: Relational trajectories in families with parental mental illness: a grounded theory approach
Source: BMC Psychol. 2020 Jul 1;8:68. doi: 10.1186/s40359-020-00432-2 (PMC7329432; doi:10.1186/s40359-020-00432-2)
Supplement: Supplementary file 1 — Additional file 1. Interview schedule. [file 40359_2020_432_MOESM1_ESM.docx]

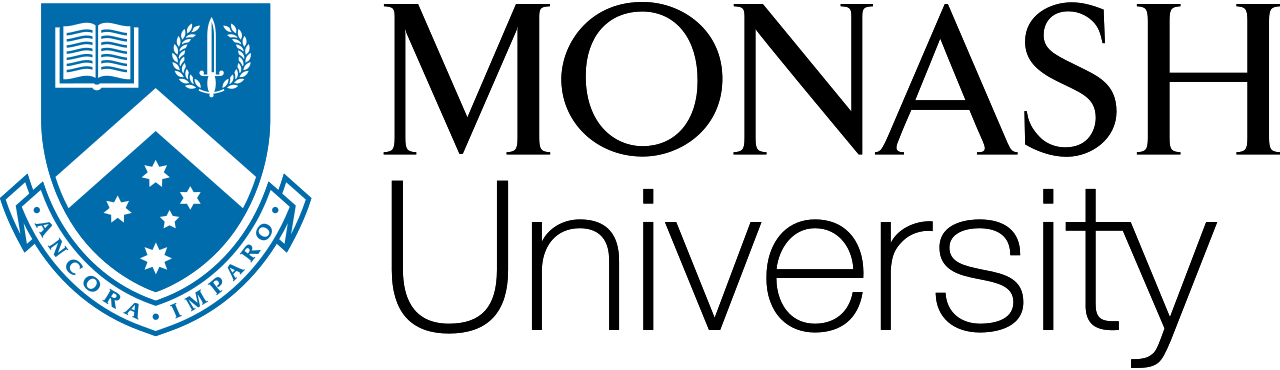


| **Interview Schedule** | |
| --- | --- |
| **Main Question** | **Follow-up Question** |
| **Section A: Nature of Parent’s Mental Illness** | |
| 1. Can you share with me about your experiences of being parented by a person with mental illness? | |
| **Section B: Experiences as a Child/Teenager** | |
| 2. Can you tell me how this affected you in your day-to-day life as a child? | |
| 3. What word comes to mind when you reflect back to your childhood and living with a parent with MI? | |
| 4. Please tell me any positive experiences, if any, associated with being parented by a person with mental illness? | |
| 5. Please tell me about any challenges, if any, associated with being parented by a person with mental illness? | 5.1 How did you manage these challenges, if at all? |
| 6. How would you describe your attachment/relationship towards the parent who had a mental illness when you were a child?  *Prompt:* Can you give me some examples of this? | 6.1 What was your attachment/relationship towards the other parent? (if they were around)  *Prompt:* Can you give me some examples of this? |
| 7. Was there someone else you could identify and rely on to take on the role parent role, when your parent with mental illness was unable to? (e.g., when you fell ill?)  *Prompt:* In what ways did you get your needs met if at all, when your parent with mental illness was unable to? | |
| 8. Were there any other significant aspects of your childhood/teenage years that were impacted by your parent’s mental illness that you would like to share? | |
| **Section C: Experiences as an Adult** | |
| Thank you for answering those questions about your childhood. Did you have anything more to add? I know some of the questions might have been challenging or may evoke strong emotions for you. Are you feeling okay at this time to proceed with this interview? **[If interviewee responds ‘yes’, read the following]:** Thank you.  The next part of the interview focuses on your adulthood experiences. Please do not hesitate to let me know should you wish to take a break or discontinue with the interview, at any time. | |
| 9. Has the quality of your relationship with your parent changed or remained the same as an adult? | 9.1 In what ways has it changed? |
| 10. What emotions or sentiments do you currently hold towards the parent with mental illness? | 10.1 How has this changed over time since you became an adult? |
| 11. In what ways has your parent’s mental illness impacted the relationships you have formed in adulthood, if at all? (e.g., with spouse, with children etc.) | |
| 12. How do you know cope with your parent’s mental illness as an adult, if at all? | 12.1 Can you elaborate or provide some examples of your coping strategies? Or what has made it difficult for you to cope?  12.2 What forms of supports have you found useful, if any? |
| **Section D: Experiences as a Parent** | |
| Thank you for answering those questions relating to your adulthood. Did you have anything more to add here? Are you feeling okay at this time to proceed with this interview?  **[If interviewee responds ‘yes’, read the following]:** Thank you. The final part of the interview focuses on your own parenting experiences. As before, please do not hesitate to let me know should you wish to take a break or discontinue with the interview, at any time.  **[If interviewee responds ‘no, read the following]:** Thank you for taking time to be a part of this interview. Would you like to continue this interview at another time of your convenience?  **[If yes, schedule another time].**  **[If no]:** I appreciate you taking time off your schedule today. The interview session will end here. I will send you a copy of the interview transcript in the coming weeks, for your comments and edits. | |
| 13. How many children do you have? And what ages are they? | |
| 14. How might you describe yourself as a parent? *Prompt:* Perhaps if you had to use an adjective or a descriptive word, how would you describe yourself as a parent?) | |
| 15. How would you describe your experiences of being a parent? | |
| 16. How would you describe the relationship you have with your own child/children? | 16.1 Does this differ for your different children?  16.2 *(If interviewee says it is different):* In what ways does it differ for each child? Could you give me some examples? |
| 17. Do you feel that your experience of being parented by a person with mental illness has impacted your own parenting role? | 17.1 What are your thoughts about this?  If yes, in what ways?  If no, why not? |
| 18. What are some positives (or strengths) you see in yourself, having become a parent? | |
| 19. What are some areas that you find more challenging as a parent? | |
| 20. Do you feel supported in your role as a parent? | 20.1 If yes, in what ways? And by whom?  20.2 If no, why not? And what support systems do you think might be helpful to you as a parent? |
| 21. How do you think you are the same as well as different from your parent? Can you give me some examples? | |
| 22. When you reflect on intergenerational patterns of behaviour and illness what do you think applies to you? And your children? | |
| 23. Do you have anything you would like to add? | |
